# Supplementary material for: Decoding the diagnostic and therapeutic potential of microbiota using pan-body pan-disease microbiomics
Source: Nat Commun. 2024 Sep 26;15:8261. doi: 10.1038/s41467-024-52598-7 (PMC11427559; doi:10.1038/s41467-024-52598-7)
Supplement: Supplementary file 1 — Supplementary Information [file 41467_2024_52598_MOESM1_ESM.pdf]

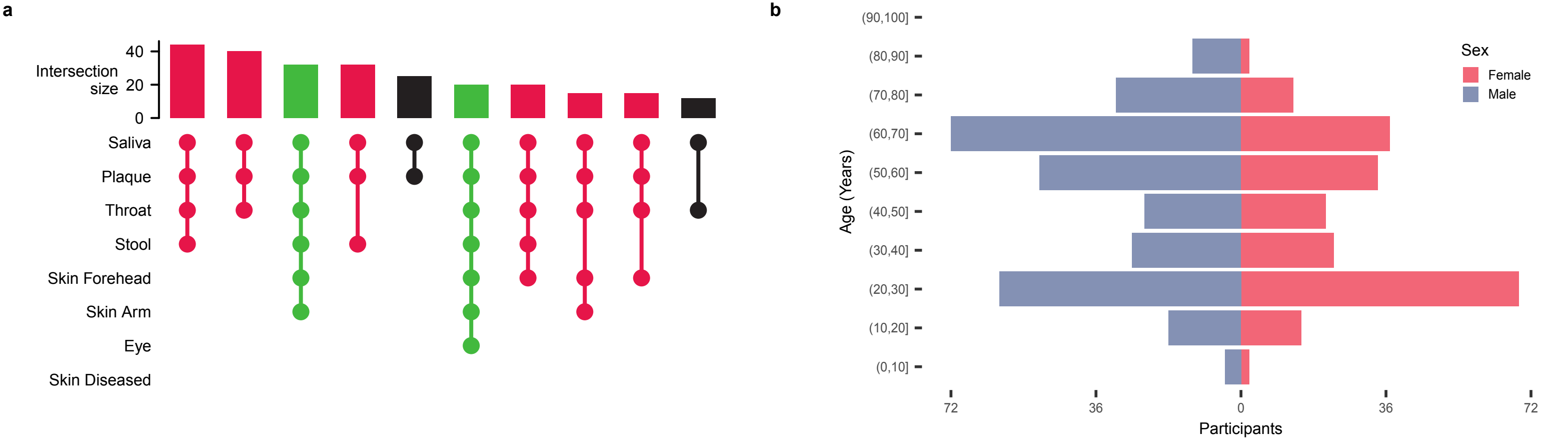

Supplementary Figure 1: a) Pruned upset plot displaying the number of patients for which the different combinations of specimen are included in the analysis. Colors in the column visually represent the quantity of specimens included, with black denoting cases where only two or fewer specimens are available, red indicating five or fewer specimens, and green signifying a high number of diverse specimens measured. **b)** Population pyramid of the included individuals.

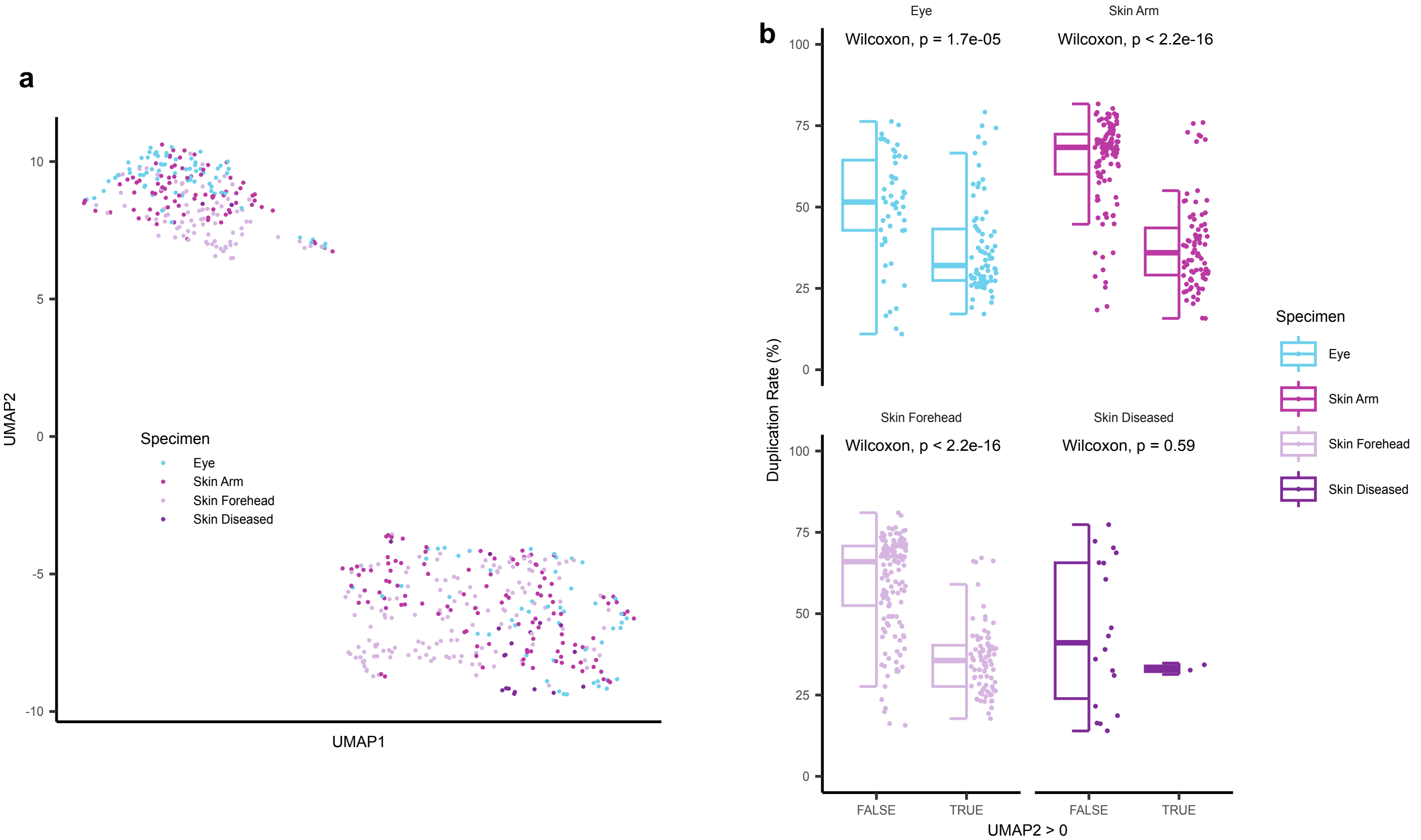

Supplementary Figure 2: a) UMAP embedding of the low input samples separates the data into two clusters. **b)** Testing the duplication rate as a potential cause for the separation of a) displayed multiple significant associations. Unadjusted p-values of two-tailed Wilcoxon rank sum test. Eye: n = 124; Skin Arm: n = 193; Skin Forehead: n = 224; Skin Diseased: n = 20. The boxplot follows a similar style to Fig 2h.

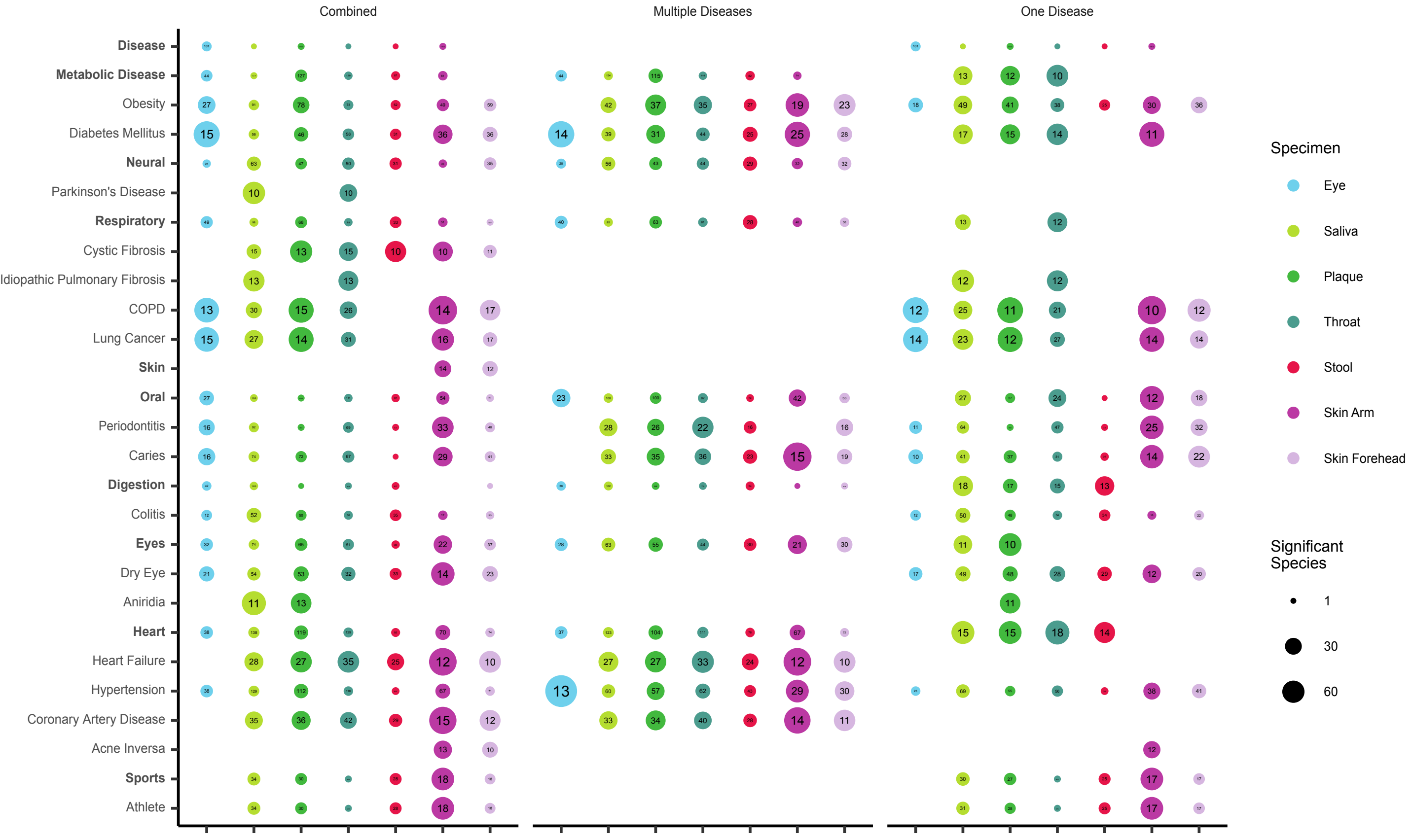

Supplementary Figure 3: Detailed version of Figure 2 e-g. Instead of filtering based on statistical significance the threshold of 0.05 after adjustement of the ANCOMBC test, associations with an absolute Cohen's d >5 are displayed.

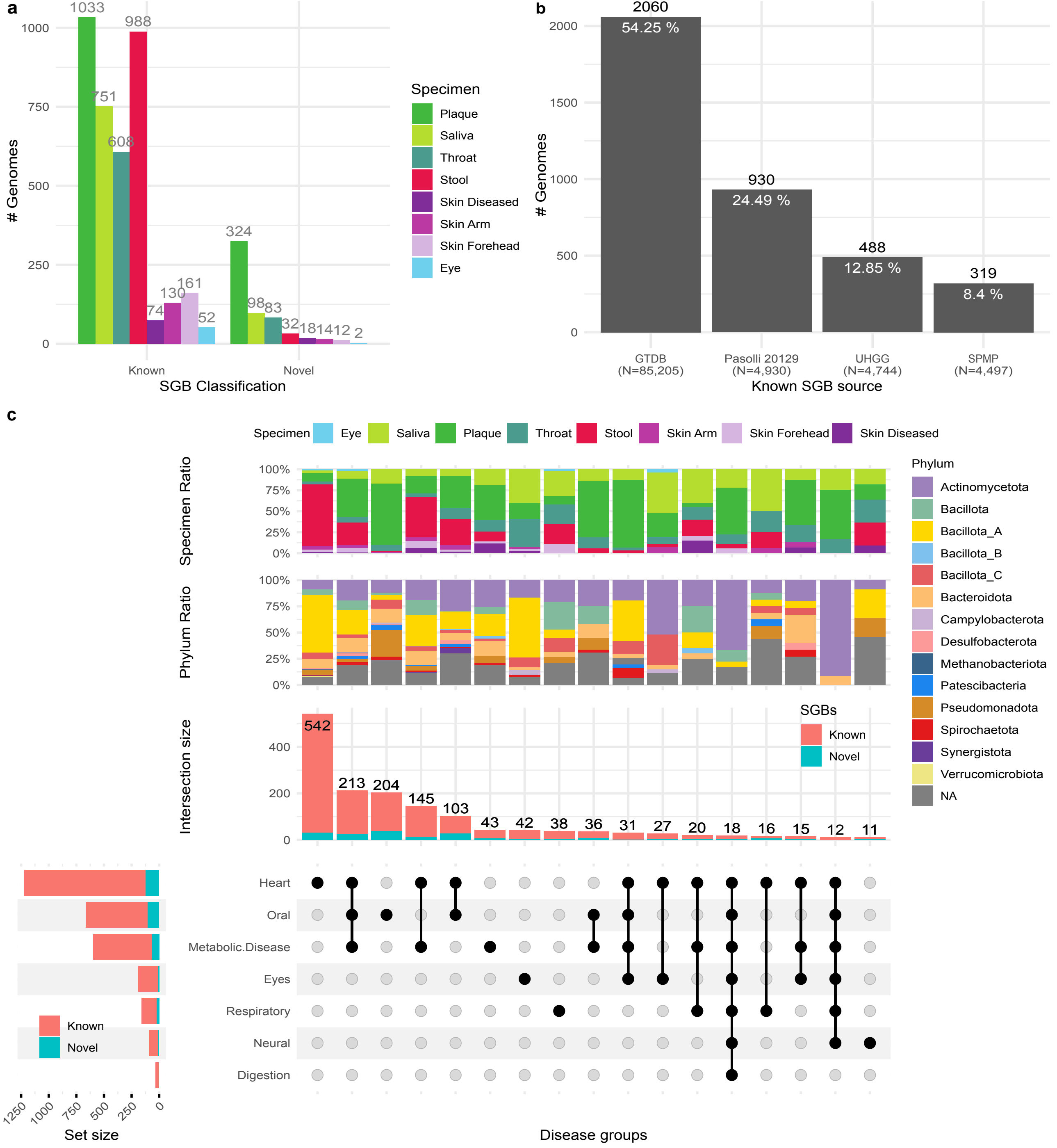

Supplementary Figure 4: a) Total number of our dereplicated SGBs visualized by specimen of the initial sample and novelty. **b)** Overlap of dereplicated SGBs with the Genome Taxonomy Database (GTDB), a study by Pasolli et al., the Singapore Platinum Metagenomes Project (SPMP), and the Unified Human Gastrointestinal Genome (UHGG) collection. Note, this plot is not adjusted for the size of the reference database. **c)** Significant SGB-disease associations computed based on coverage information. NAs indicate a lack of assignment.

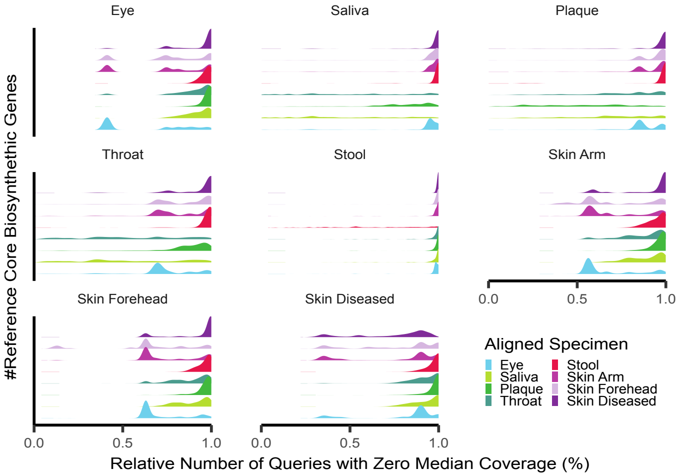

Supplementary Figure 5: Graphical representation of BGC specificity. The different panels indicate the specimen of the initial sample where the initial BGC prediction derived from. Colors indicate the specimen of the aligned samples. Reads were aligned against each core biosynthetic gene in the BGCs and median coverages were computed for each sample – gene pair.

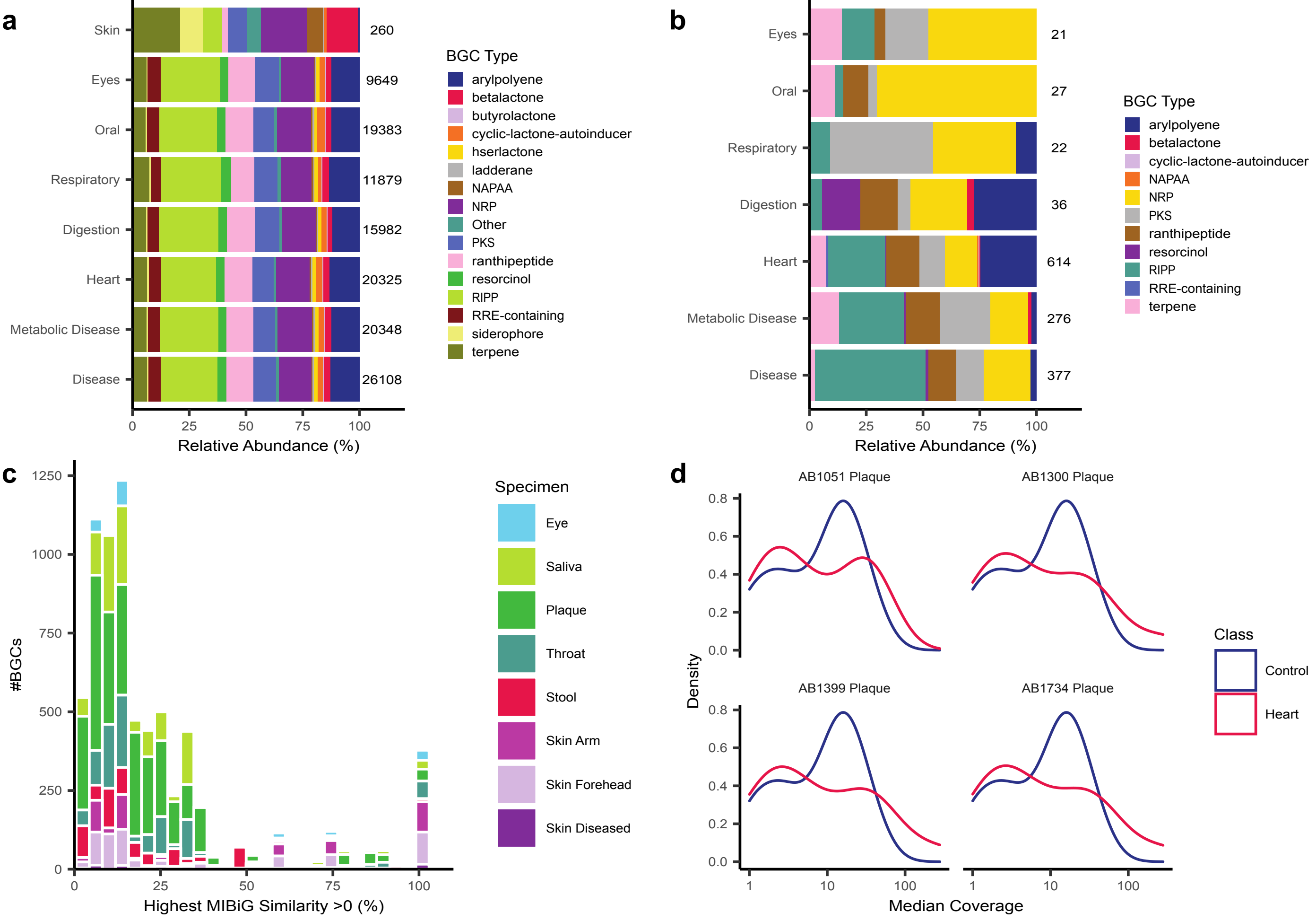

Supplementary Figure 6: a) Classes of the predicted BGCs divided by cohort of the initial sample. The total counts of predicted classes in the cohort are specified next to each row. **b)** Same as a) but focusing only on BGCs containing a significantly associated biosynthetic gene. **c)** Highest predicted non-zero similarity for all BGCs containing a significantly associated biosynthetic gene. The comparison was made against MIBiG. **d)** Median coverage distribution across tested samples for four highlighted BGC clusters that displayed a high similarity to streptin.
