# Supplementary material for: Decoding the diagnostic and therapeutic potential of microbiota using pan-body pan-disease microbiomics
Source: Nat Commun. 2024 Sep 26;15:8261. doi: 10.1038/s41467-024-52598-7 (PMC11427559; doi:10.1038/s41467-024-52598-7)
Supplement: Supplementary file 3 — Description of Additional Supplementary Files [file 41467_2024_52598_MOESM3_ESM.pdf]

Description of Additional Supplementary Files

File Name: Supplementary Data 1
Description: Number of samples included in the analysis. Information is provided for each specimen separately.

File Name: Supplementary Data 2
Description: Cohort hierarchy used for data analysis.

File Name: Supplementary Data 3
Description: Aggregated results of the differential abundance analyses performed with ANCOM-BC. The comparison was performed on patients with multiple diseases (multi), patients with only the cohort-defining disease (unique), and both (all).

File Name: Supplementary Data 4
Description: Assessment of generalized pathogenicity and commensal potential for the differentially abundant species.

File Name: Supplementary Data 5
Description: The sum of contigs surpassing different length thresholds, grouped by specimen.

File Name: Supplementary Data 6
Description: Adjusted significant levels of the Fisher's exact test comparing different antimicrobial resistance genes between specimens.

File Name: Supplementary Data 7
Description: etailed information on the species-level genome bins, their taxonomic assignment, quality, and novelty.

File Name: Supplementary Data 8
Description: Results with an adjusted p-value <1 of the differential coverage analysis of the species-level genome bins.

File Name: Supplementary Data 9
Description: Spearmen correlations of Cohen's d values between diet as a confounding factor and the different disease cohorts were computed separately for the different specimen types.
