# Supplementary material for: Decoding the diagnostic and therapeutic potential of microbiota using pan-body pan-disease microbiomics
Source: Nat Commun. 2024 Sep 26;15:8261. doi: 10.1038/s41467-024-52598-7 (PMC11427559; doi:10.1038/s41467-024-52598-7)
Supplement: Supplementary file 13 — Reporting Summary [file 41467_2024_52598_MOESM13_ESM.pdf]

nature portfolio

Corresponding author(s): Prof. Dr. Andreas Keller

Last updated by author(s): Aug 11, 2024

Reporting Summary

Nature Portfolio wishes to improve the reproducibility of the work that we publish. This form provides structure for consistency and transparency in reporting. For further information on Nature Portfolio policies, see our Editorial Policies and the Editorial Policy Checklist.

Statistics

For all statistical analyses, confirm that the following items are present in the figure legend, table legend, main text, or Methods section.

n/a	Confirmed	
☐	☒	The exact sample size (*n*) for each experimental group/condition, given as a discrete number and unit of measurement
☐	☒	A statement on whether measurements were taken from distinct samples or whether the same sample was measured repeatedly
☐	☒	The statistical test(s) used AND whether they are one- or two-sided<br>*Only common tests should be described solely by name; describe more complex techniques in the Methods section.*
☐	☒	A description of all covariates tested
☐	☒	A description of any assumptions or corrections, such as tests of normality and adjustment for multiple comparisons
☐	☒	A full description of the statistical parameters including central tendency (e.g. means) or other basic estimates (e.g. regression coefficient) AND variation (e.g. standard deviation) or associated estimates of uncertainty (e.g. confidence intervals)
☐	☒	For null hypothesis testing, the test statistic (e.g. *F*, *t*, *r*) with confidence intervals, effect sizes, degrees of freedom and *P* value noted<br>*Give P values as exact values whenever suitable.*
☐	☒	For Bayesian analysis, information on the choice of priors and Markov chain Monte Carlo settings
☐	☒	For hierarchical and complex designs, identification of the appropriate level for tests and full reporting of outcomes
☐	☒	Estimates of effect sizes (e.g. Cohen's *d*, Pearson's *r*), indicating how they were calculated

Our web collection on statistics for biologists contains articles on many of the points above.

Software and code

Policy information about availability of computer code

Data collection	Electronic case report forms were collected to be used as clinical data.
Data analysis	KneadData (version (v): 0.7.4) human sra-human-scrubber (v: 1.0.2021_05_05) fastp (v: 0.20.1) MultiQC (v: 1.11) Mash (v: 2.3) R UMAP package (v: 0.2.8) ANCOM-BC (v: 1.6.2) MetaPhlAn3 (v: 3.0.13) SPAdes (v: 3.15.4; cla: "--meta") QUAST (v: 5.0.2) MetaBAT2 (v: 2.15) dRep (v: 3.4.2) GTDB-TK (v: 2.3.0) PathoFact (commit v: 55d8240) AMRFinderPlus (v: 3.11.4) Kraken2 (v:2.1.2) Bowtie2 (v: 2.3.4.3) SAMtools idxstats (v: 1.16.1) antiSMASH (v: 6.1.1).

For manuscripts utilizing custom algorithms or software that are central to the research but not yet described in published literature, software must be made available to editors and reviewers. We strongly encourage code deposition in a community repository (e.g. GitHub). See the Nature Portfolio guidelines for submitting code & software for further information.

Data

Policy information about availability of data

All manuscripts must include a data availability statement. This statement should provide the following information, where applicable:
- Accession codes, unique identifiers, or web links for publicly available datasets
- A description of any restrictions on data availability
- For clinical datasets or third party data, please ensure that the statement adheres to our policy

The metagenomic sequencing data after removing ambient human DNA generated in this study has been deposited in the Sequencing Read Archive under the accession code PRJNA1057503.

Research involving human participants, their data, or biological material

Policy information about studies with human participants or human data. See also policy information about sex, gender (identity/presentation), and sexual orientation and race, ethnicity and racism.

Reporting on sex and gender	Sex information was self-reported. 216 Females and 299 Males were included in the study after quality control. No Sex-based analysis was performed, because it was not part of the research question.
Reporting on race, ethnicity, or other socially relevant groupings	No race or ethnicity were considered during analysis.
Population characteristics	The sample population shows a broad age distribution. Many different diseases and comorbilities are observed in the various cohorts.
Recruitment	Participants were recruited during regular medical assessments in the respective department of the Saarland University Medical Center. If an individual suffered from a disease of interest, the clinician explained the study and sampling methods. If the individual agreed, samples were taken from these patients. All participants that were medically assessed at SUMC were recruited from March 2021 to November 2023 as long as they were in line with the inclusion and exclusion criteria that were agreed upon beforehand. Therefore, there should be no selection bias.
Ethics oversight	Ethikkommission der Ärztekammer des Saarlandes, Germany

Note that full information on the approval of the study protocol must also be provided in the manuscript.

Field-specific reporting

Please select the one below that is the best fit for your research. If you are not sure, read the appropriate sections before making your selection.

☒ Life sciences ☐ Behavioural & social sciences ☐ Ecological, evolutionary & environmental sciences

For a reference copy of the document with all sections, see nature.com/documents/nr-reporting-summary-flat.pdf

Life sciences study design

All studies must disclose on these points even when the disclosure is negative.

Sample size	No power analysis was performed. Almost 3,500 samples from over 600 individuals were initially collected. A similar scale as the initial human microbiome project was achieved for WGS sequencing. (n>2300)
Data exclusions	All data exclusions are explicitly mentioned in the manuscript for transparency. Samples were always included in their entirety. Reasons for exclusion include, low initial metagenomic DNA yield, high human read contamination, and outlier behavior. Outlier behavior was assessed based on similarity to other data points generated within the study.These exclusion criteria were not pre-established. A total of >1500 samples were excluded this way due to challenges faced with skin and eye samples that display low microbial load.
Replication	A prestudy was performed and published to guarantee protocol robustness. Data has been uploaded on SRA.
Randomization	Experimental groups were aggregated based on clinical data.
Blinding	Blinding was not relevant due to a lack of intervention.

Reporting for specific materials, systems and methods

We require information from authors about some types of materials, experimental systems and methods used in many studies. Here, indicate whether each material, system or method listed is relevant to your study. If you are not sure if a list item applies to your research, read the appropriate section before selecting a response.

Materials & experimental systems

n/a	Involved in the study
☒	☐ Antibodies
☒	☐ Eukaryotic cell lines
☒	☐ Palaeontology and archaeology
☒	☐ Animals and other organisms
☐	☒ Clinical data
☒	☐ Dual use research of concern
☒	☐ Plants

Methods

n/a	Involved in the study
☒	☐ ChIP-seq
☒	☐ Flow cytometry
☒	☐ MRI-based neuroimaging

Clinical data

Policy information about clinical studies

All manuscripts should comply with the ICMJE guidelines for publication of clinical research and a completed CONSORT checklist must be included with all submissions.

Clinical trial registration	*Provide the trial registration number from ClinicalTrials.gov or an equivalent agency.*
Study protocol	*Note where the full trial protocol can be accessed OR if not available, explain why.*
Data collection	*Describe the settings and locales of data collection, noting the time periods of recruitment and data collection.*
Outcomes	*Describe how you pre-defined primary and secondary outcome measures and how you assessed these measures.*

Plants

Seed stocks	Document appears bugged; Does not apply
Novel plant genotypes	Document appears bugged; Does not apply
Authentication	Document appears bugged; Does not apply
